# Probabilistic Residual Modeling for Sensor-Based Process–Quality Fault Detection in Industrial Systems

**DOI:** 10.3390/s26134201

**Published:** 2026-07-03

**Authors:** Lirong Zhang, Xianwen Bao

**Affiliations:** School of Aeronautical Engineering, Beijing Polytechnic University, No.9, Liangshuihe 1st Street, Beijing Economic and Development Zone, Beijing 100176, China; baoxianwen@bpu.edu.cn

**Keywords:** industrial sensor data, process–quality monitoring, fault detection, probabilistic residual modeling, deep learning

## Abstract

Sensor-based process monitoring often involves both process variables and quality-related variables. These variables are usually used together to detect faults and to evaluate their effects on process quality or performance. However, most existing monitoring methods still rely on squared reconstruction residuals. This treatment assumes a fixed residual structure and may be insufficient for nonlinear industrial processes. In practice, residual variances may vary with operating conditions. Residuals from different variables may also be correlated. To address this problem, this paper proposes a probabilistic residual modeling method for process–quality fault detection. The method retains the latent-variable structure of deep variational canonical correlation analysis. It further introduces conditional residual distributions for the process side and the quality side. These distributions are parameterized by the latent operating state inferred from sensor measurements. Residual negative log-likelihoods are then used as monitoring statistics. In this way, residual abnormality is evaluated under the current operating condition. The proposed method is verified on a three-phase flow facility and a continuous stirred tank reactor process. Compared with PLS, CCA, DCCA, and DVCCA, the proposed method improves the detection of process-side disturbances and provides clearer separation between process-related and quality-related abnormal responses. Quantitatively, in the TPFF air line blockage case, the process-side statistic $J_{x}$ achieved an FDR of 82.02% with an FAR of 1.52%, compared with an FDR of 52.02% obtained by the corresponding DVCCA statistic $SPE_{x}$. In the TPFF open direct bypass case, $J_{x}$ and $J_{y}$ achieved FDRs of 90.87% and 91.07%, respectively, with FARs of 0.00%. In the CSTR coolant-temperature sensor-bias case, $J_{x}$ achieved an FDR of 88.29% with an FAR of 0.00%, while $J_{y}$ remained below its control limit, supporting process–quality fault discrimination.

## 1. Introduction

With the increasing complexity of modern industrial systems, process monitoring has become an essential technique for ensuring operational safety, product quality, and economic efficiency [1,2,3]. In industrial processes, a large number of process variable measurements are continuously collected from distributed sensors, providing valuable information for characterizing normal operating conditions and identifying abnormal events [4]. However, practical process data are usually high-dimensional, strongly correlated, nonlinear, and affected by measurement noise and varying operating conditions [5]. These characteristics make it challenging to develop reliable monitoring models that can not only detect process abnormalities but also distinguish whether a fault is related to product quality or key performance variables.

Multivariate statistical process monitoring (MSPM) methods have been widely investigated for fault detection and diagnosis [6,7]. Representative approaches include principal component analysis (PCA), partial least squares (PLS), canonical correlation analysis (CCA), and their dynamic or probabilistic extensions [8,9,10]. PCA mainly focuses on extracting dominant variance structures from process variables, while PLS and CCA are more suitable for modeling the relationship between process variables and quality-related variables. In particular, CCA aims to identify maximally correlated latent subspaces between two groups of variables, and thus provides a natural framework for process–quality correlation analysis. Nevertheless, conventional linear models are often insufficient for complex industrial processes, where nonlinear interactions and high-order dependencies are commonly observed.

To overcome the limitations of linear monitoring methods, kernel-based and deep learning-based models have received increasing attention in recent years. Kernel PCA, kernel PLS, and kernel CCA improve nonlinear feature extraction by mapping the original data into high-dimensional feature spaces [11,12,13]. However, their computational cost and scalability become significant concerns when dealing with large-scale industrial datasets. Deep neural networks provide an alternative solution by learning nonlinear representations directly from process measurements [14]. In recent years, intelligent sensor-based fault diagnosis has also been significantly advanced by deep representation learning methods. These methods aim to learn fault-sensitive representations directly from sensor signals and have shown advantages in robustness, interpretability, and adaptability under complex operating conditions. For example, the Deep Adversarial Capsule Network introduces adversarial learning and capsule representation to improve compound fault diagnosis under multidomain generalization tasks [15]. WavCapsNet combines wavelet-based signal representation with capsule networks and backward tracking to enhance the interpretability of compound fault diagnosis [16]. The Traceable Algorithm Unrolling Network integrates algorithm-unrolling ideas with sparse representation learning, providing a traceable and interpretable deep diagnostic structure [17]. The Robust Weight-shared Capsule Network improves diagnostic robustness under varying working conditions by using a weight-shared capsule architecture [18]. These studies indicate that modern sensor-based fault diagnosis is moving from purely data-driven feature extraction toward robust, interpretable, and structure-aware learning frameworks. In addition, autoencoders and variational autoencoders have been used to construct nonlinear latent variable models for process monitoring [19,20]. Deep canonical correlation analysis (DCCA) further extends CCA by learning nonlinear transformations that maximize cross-view correlation [21]. More recently, deep variational canonical correlation analysis (DVCCA) has integrated nonlinear correlation learning with probabilistic latent variable modeling, providing a generative framework for representing the shared latent structure between process variables and quality-related variables [22].

In DVCCA-based process monitoring, process measurements and quality-related variables are usually denoted as x and y, respectively [23]. A shared latent variable z is inferred from the process side and then used to reconstruct both x and y. This framework has shown promising performance in distinguishing process-related and quality-related faults. Despite this advantage, the residual modeling mechanism in existing DVCCA-based monitoring frameworks remains relatively simple. For real-valued process measurements, the reconstruction term is usually equivalent to minimizing a squared error under an implicit Gaussian observation assumption [24]. In other words, the residuals between the original measurements and their reconstructions are treated as independent or homoscedastic Gaussian noise. This assumption may be too restrictive for practical industrial processes [25]. In industrial processes, such as multiphase flow systems [26], thermal power systems [14], and chemical processes [27], faults often evolve gradually and propagate through strongly coupled physical variables. The corresponding residuals may exhibit non-Gaussian distributions and state-dependent variances. Under such circumstances, a simple squared residual may fail to accurately measure the abnormality of a sample, because the same residual magnitude may be normal under one latent operating state but abnormal under another.

This observation motivates a reconsideration of the residual monitoring mechanism in DVCCA. Instead of only evaluating the Euclidean norm of reconstruction residuals, it is more reasonable to model the conditional distribution of residuals under the current latent state. Specifically, given a latent representation z, the residuals of the process and quality spaces can be defined as $r_{x}=x-\hat{x},r_{y}=y-\hat{y}$, with $\hat{x}$ and $\hat{y}$ representing reconstructed measurements generated by the decoders. Rather than assuming fixed Gaussian residuals, the residual distributions can be described by conditional probability models $p_{\eta_{x}}\left(r_{x}|z\right)$ and $p_{\eta_{y}}\left(r_{y}|z\right)$. In this manner, process monitoring can be performed by evaluating whether the observed residual is likely to occur under the current latent operating condition. This provides a probabilistic and state-dependent interpretation of reconstruction errors. Based on this idea, this paper proposes a conditional residual-based deep variational canonical correlation analysis method, termed CR-DVCCA, for industrial process monitoring. The proposed method preserves the core advantage of DVCCA in modeling nonlinear correlations between process variables and quality-related variables, while further enhancing the residual description capability through probabilistic residual modeling. In CR-DVCCA, the latent variable is used not only to reconstruct process and quality measurements, but also to parameterize their residual distributions. Consequently, the conventional squared prediction error (SPE)-based statistics are replaced or enhanced by conditional residual negative log-likelihood indices, which can more effectively capture abnormal residual patterns under different operating states.

Compared with conventional SPE-based monitoring, the proposed CR-DVCCA has two important advantages. First, it can characterize state-dependent residual uncertainty, which is particularly useful for industrial processes where normal residual ranges may vary across operating status. Second, it can describe the coupling structure among residual variables, thereby improving the sensitivity to faults that disturb multiple physically related measurements. The main contributions of this paper are summarized as follows:

- A conditional residual-based DVCCA framework is proposed for industrial process monitoring. The proposed CR-DVCCA explicitly models the conditional residual distributions of both process variables and quality-related variables.
- New monitoring indices are developed based on conditional residual negative log-likelihoods. These indices provide probabilistic abnormality measures for the process side and the quality side, enabling effective discrimination between process-related abnormalities and quality-related faults.
- A process–quality fault discrimination strategy is established based on the proposed conditional residual monitoring indices.
- The effectiveness of the proposed method is evaluated on two industrial case studies, including the three-phase flow facility and a continuous stirred tank reactor process.

It should be noted that the proposed CR-DVCCA is related to heteroscedastic probabilistic models, probabilistic residual modeling, mixture-density monitoring, and covariance-aware anomaly detection. These methods have shown that uncertainty or covariance information can be useful for anomaly detection. However, the focus of this study is different. CR-DVCCA is developed for process–quality monitoring under the DVCCA framework, where process variables and quality-related variables need to be modeled and interpreted separately. The main novelty is therefore not only the use of a state-dependent residual covariance, but also the integration of conditional low-rank residual covariance modeling with a dual process–quality residual likelihood formulation. This formulation leads to two separate likelihood-based monitoring statistics, $J_{x}$ and $J_{y}$, which are used to distinguish process-side residual abnormalities from quality-side residual abnormalities under the current latent operating state.

The remainder of this paper is organized as follows. Section 2 briefly reviews the basic DVCCA framework and discusses the limitation of SPE-based monitoring. Section 3 presents the proposed CR-DVCCA method, including the conditional residual model, training objective, monitoring statistics, and fault discrimination strategy. Section 4 describes the offline training and online monitoring procedure. Section 5 reports the experimental results on the three-phase flow facility and the continuous stirred tank reactor case. Finally, Section 6 concludes this paper and outlines future research directions.

## 2. Preliminaries and Motivation

This section briefly introduces the problem setting and reviews the basic DVCCA framework for process monitoring. Then, the limitation of conventional SPE-based monitoring is discussed, which motivates the development of the proposed CR-DVCCA method.

### 2.1. Problem Statement

Consider an industrial process with paired process and quality-related measurements collected under normal operating conditions. Let

(1) $$X={\left[x_{1},x_{2},\ldots ,x_{n}\right]}^{T}\in R^{n\times m},\;Y={\left[y_{1},y_{2},\ldots ,y_{n}\right]}^{T}\in R^{n\times p},$$

where $x_{i}\in R^{m}$ denotes the process-variable vector of the *i*th sample, $y_{i}\in R^{p}$ denotes the corresponding quality-related or performance-variable vector, *n* is the number of normal training samples, and *m* and *p* are the numbers of process and quality-related variables, respectively. In practical industrial systems, x usually contains easily accessible operational measurements, such as pressures and temperatures. In contrast, y represents key quality or performance indicators that are directly related to product quality or equipment health.

In this study, the distinction between process variables and quality-related variables is made according to their monitoring roles and measurement characteristics. Process variables refer to online operational or sensor measurements that describe the current operating condition of the process, such as temperatures, pressures, flow rates, liquid levels, valve positions, or other continuously measured signals. These variables are usually available at relatively high sampling rates and are used to infer the latent operating state. Quality-related variables refer to product quality, key performance, or important output indicators, such as product composition, outlet concentration, efficiency, or other quality/performance indices. In industrial applications, these variables may be obtained less frequently by automatic analyzers, in-line instruments, laboratory analysis, or soft sensors. Therefore, they are treated as a separate variable block for evaluating whether a process disturbance has affected quality or performance. It should be noted that the distinction is not purely physical, since some variables may be related to both process operation and product quality. In the proposed framework, the assignment of a variable to the process block *x* or the quality-related block *y* is determined by its role in monitoring and interpretation. To avoid information duplication, the same variable is not included in both blocks in this study.

The objective of process monitoring is to learn a normal operating model from X and Y, and then determine whether a new testing sample $\left(x_{t},y_{t}\right)$ deviates from the learned normal relationship. In addition to detecting abnormal samples, it is also desirable to distinguish between process-related abnormalities and quality-related faults. Specifically, a process-related abnormality indicates that the correlation structure among process variables is damaged, while a quality-related fault implies that the abnormality has propagated to the quality-related or performance-variable space. Before model training, both process variables and quality-related variables are usually normalized using the mean and standard deviation estimated from the normal training data. The same normalization parameters are then applied to testing samples to ensure consistency between offline modeling and online monitoring.

### 2.2. Revisit of DVCCA

CCA aims to extract correlated latent representations from two groups of variables [10]. Compared with conventional CCA and deep CCA, DVCCA provides a probabilistic generative formulation for nonlinear cross-view correlation modeling [21,22,23]. In DVCCA, the dependency between x and y is represented by a shared latent variable $z\in R^{l}$, where *l* is the latent dimensionality. The generative model can be expressed as

(2) $$p_{\theta }\left(x,y,z\right)=p\left(z\right)p_{\theta_{x}}\left(x|z\right)p_{\theta_{y}}\left(y|z\right),$$

where p(z) is the prior distribution of the latent variable, and $p_{\theta_{x}}\left(x|z\right)$ and $p_{\theta_{y}}\left(y|z\right)$ are two decoder networks parameterized by $\theta_{x}$ and $\theta_{y}$, respectively. A standard Gaussian prior is usually adopted:

(3) p(z)=N(0,I).

Since the posterior distribution $p_{\theta }\left(z|x,y\right)$ is generally intractable, DVCCA introduces an amortized variational posterior to approximate the latent distribution. In process monitoring, the process-side encoder is commonly used to infer the latent representation from x:

(4) $$q_{\phi }\left(z|x\right)=N\left(\mu_{\phi }\left(x\right),\mathrm{diag}\left(\sigma_{\phi }^{2}\left(x\right)\right)\right),$$

where $\mu_{\phi }\left(x\right)$ and $\sigma_{\phi }^{2}\left(x\right)$ are the mean vector and diagonal variance vector generated by the encoder network.

For each paired sample $\left(x_{i},y_{i}\right)$, the evidence lower bound (ELBO) [20] of DVCCA can be written as

(5) $$L_{\mathrm{DVCCA}}=E_{q_{\phi }\left(z|x_{i}\right)}\left[\log p_{\theta_{x}}\left(x_{i}|z\right)+\log p_{\theta_{y}}\left(y_{i}|z\right)\right]-D_{\mathrm{KL}}\left(q_{\phi }\left(z|x_{i}\right)∥p\left(z\right)\right),$$

where $D_{\mathrm{KL}}\left(\cdot ∥\cdot \right)$ denotes the Kullback–Leibler divergence. The reparameterization trick is employed to enable gradient-based optimization:

(6) $$z=\mu_{\phi }\left(x\right)+\sigma_{\phi }\left(x\right)⊙\epsilon ,\;\epsilon ∼N\left(0,I\right),$$

where ⊙ denotes element-wise multiplication.

After training, the latent representation of a new sample can be obtained by the posterior mean:

(7) $$\hat{z}=\mu_{\phi }\left(x\right).$$

Then, the process and quality-related variables are reconstructed through the corresponding decoders:

(8) $$\hat{x}=f_{\theta_{x}}\left(\hat{z}\right),\;\hat{y}=f_{\theta_{y}}\left(\hat{z}\right).$$

where $f_{\theta_{x}}\left(\cdot \right)$ and $f_{\theta_{y}}\left(\cdot \right)$ are nonlinear mapping functions parameterized by $\theta_{x}$ and $\theta_{y}$, respectively.

In this manner, DVCCA provides a nonlinear latent variable model for simultaneously capturing the shared correlation structure between x and y and reconstructing both variable spaces.

### 2.3. SPE-Based Monitoring Statistics

In conventional DVCCA-based process monitoring, the latent representation and reconstruction errors are used to construct monitoring statistics. For the *i*th sample, the latent statistic can be defined as

(9) $$T_{i}^{2}={\left({\hat{z}}_{i}-\bar{z}\right)}^{T}S_{z}^{-1}\left({\hat{z}}_{i}-\bar{z}\right),$$

where $\bar{z}$ and $S_{z}$ are the mean vector and covariance matrix of latent representations estimated from the normal training data. When the latent variables are standardized by the prior distribution, Equation (9) can be simplified as ${\hat{z}}_{i}^{T}{\hat{z}}_{i}$.

The reconstruction residuals of the process and quality-related variables are defined as

(10) $$r_{x,i}=x_{i}-{\hat{x}}_{i},\;r_{y,i}=y_{i}-{\hat{y}}_{i}.$$

Accordingly, the SPEs of the process and quality spaces are given by

(11) $$SPE_{x,i}=∥x_{i}-{\hat{x}}_{i}{∥}_{2}^{2}={∥r_{x,i}∥}_{2}^{2},$$

and

(12) $$SPE_{y,i}=∥y_{i}-{\hat{y}}_{i}{∥}_{2}^{2}={∥r_{y,i}∥}_{2}^{2}.$$

For offline model construction, the statistics $T^{2}$, $SPE_{x}$, and $SPE_{y}$ are calculated using normal training samples. Then, their control limits are determined according to a selected confidence level. Kernel density estimation (KDE) [28] can be used to determine the thresholds:

(13) $$\psi_{T^{2}},\;\psi_{SPE_{x}},\;\psi_{SPE_{y}}.$$

For online monitoring, a testing sample is regarded as abnormal if at least one monitoring statistic exceeds its corresponding control limit. Moreover, the comparison between $SPE_{x}$ and $SPE_{y}$ provides a simple strategy for distinguishing process-related and quality-related faults.

### 2.4. Limitation of Fixed Squared Residual Monitoring

Although SPE-based monitoring is simple and effective, it relies on a restrictive residual assumption. For real-valued measurements, the reconstruction terms in DVCCA are often implemented by squared errors, which are equivalent to assuming Gaussian observation models with fixed isotropic covariance:

(14) $$p_{\theta_{x}}\left(x|z\right)=N\left(f_{\theta_{x}}\left(z\right),\sigma_{x}^{2}I\right),\;p_{\theta_{y}}\left(y|z\right)=N\left(f_{\theta_{y}}\left(z\right),\sigma_{y}^{2}I\right).$$

Under this assumption, the negative log-likelihood of the process side can be written as

(15) $$-\log p_{\theta_{x}}\left(x|z\right)=\frac{1}{2\sigma_{x}^{2}}{∥x-f_{\theta_{x}}\left(z\right)∥}_{2}^{2}+C_{x},$$

where $C_{x}$ is a constant independent of the model parameters. A similar expression holds for the quality side. Therefore, minimizing the reconstruction loss is equivalent to minimizing the squared residual.

This formulation implies that residuals with the same Euclidean norm are assigned the same abnormality level, regardless of the current operating state, variable direction, and correlation among residual components. However, this assumption may not hold in practical industrial processes [25]. First, the residual variance may be state-dependent. For example, the normal fluctuation range of temperature or flow variables may vary under different loads or operating conditions. Second, the residual components may be correlated due to physical constraints and energy balance. Third, residuals may exhibit several forms of complexity in practical industrial processes, including state-dependent variance, correlations among residual components, and, in some cases, non-Gaussian characteristics such as skewness or heavy tails. The present study mainly focuses on the first two aspects, namely heteroscedastic residual uncertainty and residual correlation, because they are directly related to the limitation of fixed squared residual monitoring. Modeling more general non-Gaussian residual distributions is beyond the scope of the current work and is discussed as a possible future extension.

As a result, the same residual magnitude may correspond to different abnormality levels under different latent operating states. A residual that is acceptable under one operating condition may indicate a fault under another condition. Conversely, a large residual in a high-uncertainty operating region may be incorrectly identified as a fault if only the squared norm is considered. These issues can lead to missed detections, false alarms, and insufficient fault interpretation.

## 3. CR-DVCCA Algorithm

The basic idea of CR-DVCCA is to preserve the nonlinear process–quality correlation modeling capability of DVCCA, while replacing the fixed squared residual monitoring mechanism with a conditional residual probability model. In this way, the abnormality of a reconstruction residual is evaluated according to its likelihood under the current latent operating state, rather than only according to its Euclidean magnitude.

### 3.1. Model Structure

Given paired process and quality-related measurements (x,y), CR-DVCCA assumes that their nonlinear dependency can be represented by a shared latent variable $z\in R^{l}$. Similar to DVCCA, the latent variable is assigned a standard Gaussian prior p(z)=N(0,I). As presented in Equation (4), the posterior distribution of z is approximated by an encoder network $q_{\phi }\left(z|x\right)=N\left(\mu_{\phi }\left(x\right),\mathrm{diag}\left(\sigma_{\phi }^{2}\left(x\right)\right)\right)$. The latent variable is then used to reconstruct the process variables and quality-related variables through two decoder networks $\hat{x}=f_{\theta_{x}}\left(z\right),\hat{y}=f_{\theta_{y}}\left(z\right)$. The reconstruction residuals are defined in Equation (10). Different from DVCCA, which usually measures $r_{x}$ and $r_{y}$ by squared Euclidean norms, CR-DVCCA explicitly models their conditional probability distributions:

(16) $$r_{x}\left|z∼p_{\eta_{x}}\left(r_{x}|z\right),\;r_{y}\right|z∼p_{\eta_{y}}\left(r_{y}|z\right),$$

where $p_{\eta_{x}}\left(r_{x}|z\right)$ and $p_{\eta_{y}}\left(r_{y}|z\right)$ denote the conditional residual distributions of the process side and quality side, respectively. The parameters $\eta_{x}$ and $\eta_{y}$ are learned from normal operating data.

Accordingly, the generative model of CR-DVCCA can be written as

(17) $$p_{\Theta }\left(x,y,z\right)=p\left(z\right)p_{\eta_{x}}\left(x-f_{\theta_{x}}\left(z\right)|z\right)p_{\eta_{y}}\left(y-f_{\theta_{y}}\left(z\right)|z\right),$$

where $\Theta =\{\theta_{x},\theta_{y},\eta_{x},\eta_{y}\}$ denotes the set of generative model parameters.

Equation (17) indicates that the latent variable z is used not only to reconstruct the mean values of x and y, but also to characterize the residual uncertainty under the current operating state. In this formulation, the decoder outputs $f_{\theta_{x}}\left(z\right)$ and $f_{\theta_{y}}\left(z\right)$ are treated as the conditional means of the process and quality observations, respectively. Therefore, modeling the residual distributions is equivalent to modeling the observation distributions with state-dependent covariance. Specifically, if $r_{x}=x-f_{\theta_{x}}\left(z\right)$ and $r_{x}|z∼N\left(0,\Sigma_{x}\left(z\right)\right)$, then the induced observation likelihood is $x|z∼N(f_{\theta_{x}}\left(z\right),\Sigma_{x}\left(z\right))$. This equivalence holds because the transformation from *x* to $r_{x}$ is a translation with unit Jacobian determinant. Similarly, $r_{y}|z∼N\left(0,\Sigma_{y}\left(z\right)\right)$ induces $y|z∼N(f_{\theta_{y}}\left(z\right),\Sigma_{y}\left(z\right))$. Thus, the residual likelihood used in CR-DVCCA can also be interpreted as an observation likelihood with conditional mean and state-dependent covariance.

### 3.2. Conditional Residual Distribution

For industrial process data, the residuals of different variables are often correlated due to physical constraints or energy balance relationships. Meanwhile, the residual uncertainty may vary with the latent operating condition. To capture these characteristics while maintaining computational efficiency, this study adopts a conditional low-rank Gaussian residual model. The conditional Gaussian assumption is adopted here as a tractable probabilistic approximation rather than as a claim that all industrial residuals are exactly Gaussian. Compared with the fixed isotropic Gaussian assumption underlying SPE-based monitoring, the proposed conditional Gaussian model allows the residual covariance to depend on the latent operating state and further captures correlations among residual variables through the low-rank term. Therefore, the current formulation mainly improves the modeling of state-dependent residual uncertainty and covariance structure. More flexible residual likelihoods, such as conditional Student’s t-distributions, Gaussian mixture residual models, or normalizing-flow-based residual models, could be incorporated in future work to describe heavy-tailed or skewed residual distributions more explicitly.

For the process side, the conditional residual distribution is defined as

(18) $$p_{\eta_{x}}\left(r_{x}|z\right)=N\left(0,\Sigma_{x}\left(z\right)\right),$$

where the covariance matrix $\Sigma_{x}\left(z\right)$ is state-dependent. Similarly, for the quality side,

(19) $$p_{\eta_{y}}\left(r_{y}|z\right)=N\left(0,\Sigma_{y}\left(z\right)\right).$$

The conditional covariance matrices are parameterized as

(20) $$\Sigma_{x}\left(z\right)=D_{x}\left(z\right)+B_{x}\left(z\right)B_{x}^{T}\left(z\right),$$

and

(21) $$\Sigma_{y}\left(z\right)=D_{y}\left(z\right)+B_{y}\left(z\right)B_{y}^{T}\left(z\right),$$

where $D_{x}\left(z\right)\in R^{m\times m}$ and $D_{y}\left(z\right)\in R^{p\times p}$ are diagonal positive definite matrices, and $B_{x}\left(z\right)\in R^{m\times k_{x}}$ and $B_{y}\left(z\right)\in R^{p\times k_{y}}$ are low-rank factor matrices. Here, $k_{x}\ll m$ and $k_{y}\ll p$ are the predefined residual ranks.

The diagonal terms are used to describe the variable-wise residual variances:

(22) $$D_{x}\left(z\right)=\mathrm{diag}\left(\mathrm{softplus}\left(d_{x}\left(z\right)\right)+\epsilon \right),$$

(23) $$D_{y}\left(z\right)=\mathrm{diag}\left(\mathrm{softplus}\left(d_{y}\left(z\right)\right)+\epsilon \right),$$

where $d_{x}\left(z\right)$ and $d_{y}\left(z\right)$ are neural network outputs, softplus(·) is used to ensure positivity, and ϵ is a small positive constant for numerical stability.

The low-rank terms $B_{x}\left(z\right)B_{x}^{T}\left(z\right)$ and $B_{y}\left(z\right)B_{y}^{T}\left(z\right)$ are introduced to capture the correlation structure among residual variables. Compared with a full covariance matrix, the low-rank structure significantly reduces the number of parameters and improves numerical stability. Compared with a diagonal covariance matrix, it allows CR-DVCCA to model coupled residual variations among physically related variables. Under the Gaussian residual assumption, the negative log-likelihood of the process-side residual is

(24) $$-\log p_{\eta_{x}}\left(r_{x}|z\right)=\frac{1}{2}r_{x}^{T}\Sigma_{x}^{-1}\left(z\right)r_{x}+\frac{1}{2}\log\left|\Sigma_{x}\left(z\right)\right|+\frac{m}{2}\log\left(2\pi \right).$$

Similarly, the quality-side residual negative log-likelihood is

(25) $$-\log p_{\eta_{y}}\left(r_{y}|z\right)=\frac{1}{2}r_{y}^{T}\Sigma_{y}^{-1}\left(z\right)r_{y}+\frac{1}{2}\log\left|\Sigma_{y}\left(z\right)\right|+\frac{p}{2}\log\left(2\pi \right).$$

It can be observed that the residual abnormality is determined by both the Mahalanobis distance term and the log-determinant term. The first term evaluates the residual deviation considering the conditional covariance structure, while the second term penalizes the uncertainty volume of the residual distribution. Therefore, the model cannot simply increase the covariance to reduce the residual distance, because an enlarged covariance will also increase the log-determinant penalty.

### 3.3. Variational Inference and Training Objective

Since the true posterior distribution of the latent variable is generally intractable, the variational posterior in Equation (4) is used for approximate inference. For each paired training sample $\left(x_{i},y_{i}\right)$, the latent variable is sampled using the reparameterization trick:

(26) $$z_{i}=\mu_{\phi }\left(x_{i}\right)+\sigma_{\phi }\left(x_{i}\right)⊙\epsilon_{i},\;\epsilon_{i}∼N\left(0,I\right).$$

The evidence lower bound of CR-DVCCA is formulated as

(27) $$L_{\mathrm{CR}-\mathrm{DVCCA}}=E_{q_{\phi }\left(z|x\right)}\left[\log p_{\eta_{x}}\left(r_{x}|z\right)+\lambda_{y}\log p_{\eta_{y}}\left(r_{y}|z\right)\right]-\beta D_{\mathrm{KL}}\left(q_{\phi }\left(z|x\right)∥p\left(z\right)\right),$$

where $\lambda_{y}$ is a weighting coefficient for the quality-side residual likelihood, and β controls the strength of the KL regularization term. Equivalently, the training objective can be written as the following minimization problem:

(28) $$J_{\mathrm{CR}-\mathrm{DVCCA}}=-E_{q_{\phi }\left(z|x\right)}\left[\log p_{\eta_{x}}\left(r_{x}|z\right)+\lambda_{y}\log p_{\eta_{y}}\left(r_{y}|z\right)\right]+\beta D_{\mathrm{KL}}\left(q_{\phi }\left(z|x\right)∥p\left(z\right)\right).$$

The two weighting parameters $\lambda_{y}$ and β affect the balance of different terms in the training objective. The coefficient $\lambda_{y}$ controls the relative contribution of the quality-side residual likelihood. When $\lambda_{y}$ is too small, the model tends to focus mainly on the process-side residual distribution, which may weaken the sensitivity of the quality-side monitoring statistic. In contrast, an excessively large $\lambda_{y}$ may overemphasize the quality-side residual term and reduce the balance between process-side and quality-side residual modeling. The parameter β controls the strength of the KL regularization. A small β may result in insufficient regularization of the latent space, whereas a large β may over-constrain the latent representation and lead to underfitting. When $\lambda_{y}=1$ and β=1, Equation (27) corresponds to the standard ELBO associated with the probabilistic model in Equation (17). In practical monitoring applications, $\lambda_{y}$ and β are introduced to obtain a weighted ELBO-type objective. Equation (28) should be interpreted as a weighted negative ELBO or β-regularized training objective rather than an unmodified standard ELBO when $\lambda_{y}\neq 1$ or β≠1. Therefore, these two parameters should be selected to balance reconstruction accuracy, residual likelihood modeling, and latent-space regularization.

For a diagonal Gaussian variational posterior and a standard Gaussian prior, the KL divergence has a closed-form expression:

(29) $$D_{\mathrm{KL}}\left(q_{\phi }\left(z|x\right)∥p\left(z\right)\right)=\frac{1}{2}\sum_{j=1}^{l}\left(\mu_{j}^{2}+\sigma_{j}^{2}-\log\sigma_{j}^{2}-1\right),$$

where $\mu_{j}$ and $\sigma_{j}^{2}$ are the *j*th elements of $\mu_{\phi }\left(x\right)$ and $\sigma_{\phi }^{2}\left(x\right)$, respectively.

During training, the network parameters ϕ, $\theta_{x}$, $\theta_{y}$, $\eta_{x}$, and $\eta_{y}$ are optimized jointly by stochastic gradient descent methods. The expectation in Equation (28) can be approximated using one or several Monte Carlo samples of z. In practice, using one latent sample for each input is usually sufficient for efficient training.

### 3.4. Relationship with SPE-Based DVCCA

The proposed CR-DVCCA can be regarded as a probabilistic extension of SPE-based DVCCA. If the conditional residual covariance matrices are reduced to fixed isotropic covariance matrices,

(30) $$\Sigma_{x}\left(z\right)=\sigma_{x}^{2}I,\;\Sigma_{y}\left(z\right)=\sigma_{y}^{2}I,$$

then the process-side residual negative log-likelihood becomes

(31) $$-\log p_{\eta_{x}}\left(r_{x}|z\right)=\frac{1}{2\sigma_{x}^{2}}{∥r_{x}∥}_{2}^{2}+C_{x},$$

where $C_{x}$ is a constant independent of the residual. Similarly,

(32) $$-\log p_{\eta_{y}}\left(r_{y}|z\right)=\frac{1}{2\sigma_{y}^{2}}{∥r_{y}∥}_{2}^{2}+C_{y}.$$

Therefore, minimizing the residual negative log-likelihood is equivalent to minimizing $SPE_{x}$ and $SPE_{y}$ under the fixed isotropic Gaussian assumption. This shows that SPE-based DVCCA is a special case of CR-DVCCA. Furthermore, if $\Sigma_{x}\left(z\right)$ and $\Sigma_{y}\left(z\right)$ are diagonal but state-dependent, CR-DVCCA becomes a conditional weighted SPE model. In this case, each residual component is weighted according to its state-dependent variance. If the low-rank terms are retained, CR-DVCCA further captures the coupling structure among residual variables. Hence, the proposed method provides a gradual extension from fixed SPE monitoring to state-dependent and correlation-aware residual monitoring.

### 3.5. Monitoring Statistics of CR-DVCCA

Compared with heteroscedastic VAE models, CR-DVCCA does not only introduce state-dependent observation uncertainty for reconstruction. Instead, it explicitly constructs two conditional residual distributions for the process side and the quality side within a shared latent process–quality correlation model. Compared with general probabilistic residual modeling or mixture-density monitoring methods, CR-DVCCA uses the latent operating state inferred from process measurements to parameterize the residual covariance and further separates the residual likelihoods of *x* and *y*. Compared with covariance-aware anomaly detection methods based on a fixed or global covariance matrix, the proposed method adopts a state-dependent diagonal-plus-low-rank covariance structure, allowing the residual uncertainty and residual correlations to vary with the operating condition. Therefore, CR-DVCCA provides not only a probabilistic residual abnormality measure, but also a process–quality-oriented monitoring structure for fault discrimination.

After the CR-DVCCA model is trained using normal operating data, monitoring statistics can be constructed for online process monitoring. To avoid randomness during online monitoring, the posterior mean is used as the deterministic latent representation:

(33) $$\hat{z}=\mu_{\phi }\left(x\right).$$

The latent-space monitoring statistic is defined as

(34) $$J_{z}={\left(\hat{z}-\bar{z}\right)}^{T}S_{z}^{-1}\left(\hat{z}-\bar{z}\right),$$

where $\bar{z}$ and $S_{z}$ are the mean vector and covariance matrix of the latent representations calculated from the normal training data. If the latent variables are sufficiently regularized by the standard Gaussian prior, $J_{z}$ can be simplified as ${\hat{z}}^{T}\hat{z}$.

The process-side residual is calculated as

(35) $${\hat{r}}_{x}=x-f_{\theta_{x}}\left(\hat{z}\right),$$

and its conditional residual monitoring statistic is defined by the negative log-likelihood:

(36) $$J_{x}=-\log p_{\eta_{x}}\left({\hat{r}}_{x}|\hat{z}\right).$$

According to Equation (24), $J_{x}$ can be calculated as

(37) $$J_{x}=\frac{1}{2}{\hat{r}}_{x}^{T}\Sigma_{x}^{-1}\left(\hat{z}\right){\hat{r}}_{x}+\frac{1}{2}\log\left|\Sigma_{x}\left(\hat{z}\right)\right|+\frac{m}{2}\log\left(2\pi \right).$$

Similarly, the quality-side residual is

(38) $${\hat{r}}_{y}=y-f_{\theta_{y}}\left(\hat{z}\right),$$

and the corresponding monitoring statistic is

(39) $$J_{y}=-\log p_{\eta_{y}}\left({\hat{r}}_{y}|\hat{z}\right),$$

which can be explicitly written as

(40) $$J_{y}=\frac{1}{2}{\hat{r}}_{y}^{T}\Sigma_{y}^{-1}\left(\hat{z}\right){\hat{r}}_{y}+\frac{1}{2}\log\left|\Sigma_{y}\left(\hat{z}\right)\right|+\frac{p}{2}\log\left(2\pi \right).$$

The statistic $J_{z}$ measures the deviation of the sample from the normal latent space. The statistic $J_{x}$ evaluates whether the process-side residual is abnormal under the current latent operating condition. The statistic $J_{y}$ evaluates whether the quality-side residual is abnormal under the same latent state. Compared with $SPE_{x}$ and $SPE_{y}$, $J_{x}$ and $J_{y}$ provide state-dependent probabilistic abnormality measures.

## 4. Process Monitoring Procedure

**Figure 1:**
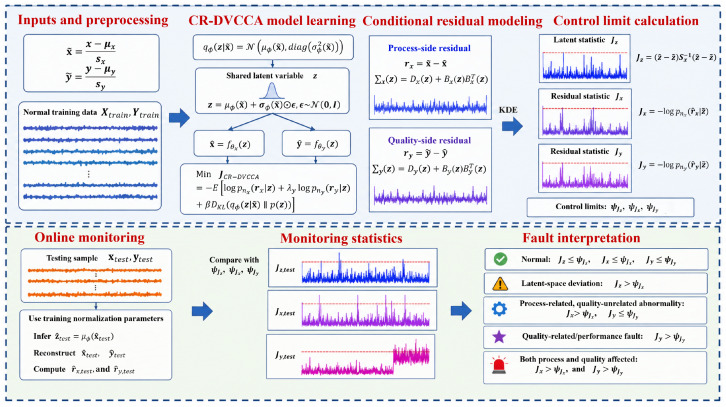
Flowchart of the proposed CR-DVCCA.

This section presents the process monitoring procedure based on the proposed CR-DVCCA method. As illustrated in Figure 1, the whole framework consists of two main stages: offline training and online monitoring. In the offline training stage, normal operating data are used to train the CR-DVCCA model and determine the control limits of the monitoring statistics. In the online monitoring stage, real-time samples are projected into the learned latent space, and the conditional residual monitoring statistics are calculated for abnormality detection and fault interpretation.

### 4.1. Offline Training

Suppose that the normal operating data are collected as

(41) $$X_{train}={\left[x_{1},x_{2},\ldots ,x_{n}\right]}^{T}\in R^{n\times m},\;Y_{train}={\left[y_{1},y_{2},\ldots ,y_{n}\right]}^{T}\in R^{n\times p},$$

where $x_{i}$ denotes the process-variable vector, $y_{i}$ denotes the quality-related or performance-variable vector, and *n* is the number of normal training samples.

Before model training, all variables are standardized using the mean and standard deviation estimated from the normal training data:

(42) $${\tilde{x}}_{i,j}=\frac{x_{i,j}-\mu_{x,j}}{s_{x,j}},\;{\tilde{y}}_{i,j}=\frac{y_{i,j}-\mu_{y,j}}{s_{y,j}},$$

where $\mu_{x,j}$ and $s_{x,j}$ are the mean and standard deviation of the *j*th process variable, while $\mu_{y,j}$ and $s_{y,j}$ are those of the *j*th quality-related variable. The same normalization parameters are stored and used for online testing samples.

In practical deployment, the normalization parameters used in Equation (42) should be stored together with the trained monitoring model and applied consistently during online monitoring. The use of the mean and standard deviation estimated from normal training data is adopted in this study to ensure consistency between offline modeling and online monitoring. Alternatively, if reliable engineering references are available, the variables can also be normalized using fixed sensor measurement ranges, design operating ranges, or engineering lower and upper limits. Such range-based normalization may reduce dependence on a specific training dataset and can be convenient for plant implementation. However, the same normalization rule must be used consistently during model training, control-limit determination, and online monitoring. If the normalization reference is changed, the model parameters and control limits should be retrained or recalibrated accordingly.

The standardized normal data are then used to train the CR-DVCCA model by minimizing the objective function in Equation (28). After training, the posterior mean is used as the deterministic latent representation:

(43) $${\hat{z}}_{i}=\mu_{\phi }\left({\tilde{x}}_{i}\right).$$

The reconstructed measurements are obtained by

(44) $${\hat{x}}_{i}=f_{\theta_{x}}\left({\hat{z}}_{i}\right),\;{\hat{y}}_{i}=f_{\theta_{y}}\left({\hat{z}}_{i}\right).$$

Then, the process-side and quality-side residuals are calculated as

(45) $${\hat{r}}_{x,i}={\tilde{x}}_{i}-{\hat{x}}_{i},\;{\hat{r}}_{y,i}={\tilde{y}}_{i}-{\hat{y}}_{i}.$$

Based on the trained CR-DVCCA model, the monitoring statistics $J_{z}$, $J_{x}$, and $J_{y}$ are computed for all normal training samples. Their control limits are determined from the empirical distributions of the corresponding statistics using KDE. For a generic monitoring statistic *J* with normal statistic values $J_{1},J_{2},\ldots ,J_{n}$, its probability density function can be estimated as

(46) $$\hat{f}\left(J\right)=\frac{1}{nh}\sum_{i=1}^{n}K\left(\frac{J-J_{i}}{h}\right),$$

where K(·) is the kernel function and *h* is the bandwidth. In this study, a Gaussian kernel is adopted, and the bandwidth is selected using Silverman’s rule-of-thumb. The control limit $\psi_{J}$ is determined by

(47) $$\int_{-\infty }^{\psi_{J}}\hat{f}\left(J\right)dJ=1-\alpha ,$$

where α is the significance level. In the experiments, the confidence level is set to (1−α = 95%), i.e., (α = 0.05), for all monitoring statistics. Therefore, the control limits of CR-DVCCA are obtained as

(48) $$\psi_{J_{z}},\;\psi_{J_{x}},\;\psi_{J_{y}}.$$

To improve reproducibility and avoid using testing information during threshold determination, the KDE-based control limits are estimated only from normal data. The testing samples are not involved in either model training or threshold determination. In the present implementation, the normal samples used for threshold calibration are separated from the fault testing samples. When sufficient normal data are available, an independent normal validation subset can be used to further reduce the risk of threshold overfitting.

### 4.2. Online Monitoring

For a new testing sample $\left(x_{test},y_{test}\right)$, the same standardization parameters obtained from the normal training data are first applied:

(49) $${\tilde{x}}_{test,j}=\frac{x_{test,j}-\mu_{x,j}}{s_{x,j}},\;{\tilde{y}}_{test,j}=\frac{y_{test,j}-\mu_{y,j}}{s_{y,j}}.$$

Then, the latent representation is inferred by the encoder:

(50) $${\hat{z}}_{test}=\mu_{\phi }\left({\tilde{x}}_{test}\right).$$

The reconstructed process and quality-related variables are calculated as

(51) $${\hat{x}}_{test}=f_{\theta_{x}}\left({\hat{z}}_{test}\right),\;{\hat{y}}_{test}=f_{\theta_{y}}\left({\hat{z}}_{test}\right).$$

The corresponding residuals are

(52) $${\hat{r}}_{x,test}={\tilde{x}}_{test}-{\hat{x}}_{test},\;{\hat{r}}_{y,test}={\tilde{y}}_{test}-{\hat{y}}_{test}.$$

The online monitoring statistics are then computed as

(53) $$J_{z,test}={\left({\hat{z}}_{test}-\bar{z}\right)}^{T}S_{z}^{-1}\left({\hat{z}}_{test}-\bar{z}\right),$$

(54) $$J_{x,test}=-\log p_{\eta_{x}}\left({\hat{r}}_{x,test}|{\hat{z}}_{test}\right),$$

and

(55) $$J_{y,test}=-\log p_{\eta_{y}}\left({\hat{r}}_{y,test}|{\hat{z}}_{test}\right).$$

The testing sample is considered normal if all monitoring statistics are within their corresponding control limits:

(56) $$J_{z,test}\leq \psi_{J_{z}},\;J_{x,test}\leq \psi_{J_{x}},\;J_{y,test}\leq \psi_{J_{y}}.$$

Otherwise, an abnormal condition is detected.

For industrial processes, isolated alarms may be caused by measurement noise or short-term disturbances. Therefore, in practical implementation, a consecutive alarm rule can be adopted. Specifically, a confirmed alarm is triggered only when at least one monitoring statistic exceeds its corresponding control limit for $N_{a}$ consecutive samples. The parameter $N_{a}$ controls the trade-off between false alarm suppression and detection delay. A larger $N_{a}$ can reduce isolated false alarms, but it may also delay fault detection. In contrast, a smaller $N_{a}$ provides faster detection but is more sensitive to noise. In the reported case studies, the FDR and FAR values were calculated using single-sample threshold exceedance, which is equivalent to $N_{a}=1$, to avoid introducing additional alarm delay and to ensure fair comparison among different monitoring methods. In practical deployment, $N_{a}$ can be selected according to the sampling interval, process noise level, and alarm management requirements.

The overall process monitoring framework based on CR-DVCCA is summarized in Algorithm 1. For industrial processes, isolated alarms may be caused by measurement noise or short-term disturbances. Therefore, in practical implementation, a consecutive alarm rule can be adopted. Specifically, an alarm is triggered only when at least one monitoring statistic exceeds its corresponding control limit for $N_{a}$ consecutive samples. This strategy improves online monitoring robustness without changing the basic CR-DVCCA monitoring mechanism.
**Algorithm 1** The proposed process monitoring framework based on CR-DVCCA.  1:**Inputs:** Normal training data $X_{train}$ and $Y_{train}$, real-time testing data $x_{test}$ and $y_{test}$;  2:**Offline Training:**  3:Standardize $X_{train}$ and $Y_{train}$ according to Equation (42);  4:Train the CR-DVCCA model by minimizing the objective function in Equation (28), and obtain the model parameters ϕ, $\theta_{x}$, $\theta_{y}$, $\eta_{x}$, and $\eta_{y}$;  5:For i=1 to *n* do  6:    Compute the latent representation ${\hat{z}}_{i}=\mu_{\phi }\left({\tilde{x}}_{i}\right)$;  7:    Calculate reconstructed measurements ${\hat{x}}_{i}=f_{\theta_{x}}\left({\hat{z}}_{i}\right)$ and ${\hat{y}}_{i}=f_{\theta_{y}}\left({\hat{z}}_{i}\right)$;  8:    Calculate residuals ${\hat{r}}_{x,i}={\tilde{x}}_{i}-{\hat{x}}_{i}$ and ${\hat{r}}_{y,i}={\tilde{y}}_{i}-{\hat{y}}_{i}$;  9:    Estimate conditional covariance matrices $\Sigma_{x}\left({\hat{z}}_{i}\right)$ and $\Sigma_{y}\left({\hat{z}}_{i}\right)$;10:End For11:Calculate the latent mean $\bar{z}$ and covariance matrix $S_{z}$ using all normal latent representations;12:Compute monitoring indices $J_{z}$, $J_{x}$, and $J_{y}$ of the training set according to Equations (53)–(55);13:Calculate the corresponding control limits $\psi_{J_{z}}$, $\psi_{J_{x}}$, and $\psi_{J_{y}}$ according to the KDE method in Equations (46) and (47);14:**Online Monitoring:**15:Standardize the testing sample $x_{test}$ and $y_{test}$ using the training mean and standard deviation;16:Compute the latent representation ${\hat{z}}_{test}=\mu_{\phi }\left({\tilde{x}}_{test}\right)$;17:Calculate reconstructed measurements ${\hat{x}}_{test}=f_{\theta_{x}}\left({\hat{z}}_{test}\right)$ and ${\hat{y}}_{test}=f_{\theta_{y}}\left({\hat{z}}_{test}\right)$;18:Calculate residuals ${\hat{r}}_{x,test}={\tilde{x}}_{test}-{\hat{x}}_{test}$ and ${\hat{r}}_{y,test}={\tilde{y}}_{test}-{\hat{y}}_{test}$;19:Compute testing indices $J_{z,test}$, $J_{x,test}$, and $J_{y,test}$ according to Equations (53)–(55);20:The process is treated as normal if $J_{z,test}\leq \psi_{J_{z}}$, $J_{x,test}\leq \psi_{J_{x}}$, and $J_{y,test}\leq \psi_{J_{y}}$;21:If $J_{z,test}>\psi_{J_{z}}$, the testing sample deviates from the normal latent operating space and an out-of-distribution operating state or latent-space deviation is indicated;22:If $J_{x,test}>\psi_{J_{x}}$ and $J_{y,test}\leq \psi_{J_{y}}$, the fault can be regarded as a process-related but quality-unrelated abnormality;23:If $J_{y,test}>\psi_{J_{y}}$, a quality-related or performance-related fault is observed;24:If $J_{x,test}>\psi_{J_{x}}$ and $J_{y,test}>\psi_{J_{y}}$, the fault affects both the process-variable space and the quality-related variable space.

### 4.3. Fault Interpretation Strategy

The proposed CR-DVCCA method provides three monitoring statistics with different meanings. The latent statistic $J_{z}$ reflects whether the current operating state deviates from the normal latent space. The process-side statistic $J_{x}$ evaluates whether the process-side conditional residual is abnormal under the current latent state. The quality-side statistic $J_{y}$ evaluates whether the quality-side or performance-side residual is abnormal under the same latent state. Based on these statistics, the fault interpretation rules can be summarized in Table 1.

From an engineering perspective, the proposed monitoring statistics can be interpreted as three complementary health indicators rather than purely mathematical quantities. The latent statistic $J_{z}$ indicates whether the current sample is located outside the normal operating region learned from historical normal data. Therefore, an alarm in $J_{z}$ can be explained to an operator as the current operating state is different from the normal operating patterns. The process-side statistic $J_{x}$ evaluates whether the process-variable measurements are mutually consistent under the current latent operating state. An alarm in $J_{x}$ suggests that the process sensors or process-variable relationships show an abnormal residual pattern. The quality-side statistic $J_{y}$ evaluates whether the quality-related or performance-related variables are consistent with the expected process–quality relationship. An alarm in $J_{y}$ therefore indicates that the abnormality may have affected product quality or key performance. The corresponding control limits can be regarded as empirical boundaries of normal behavior estimated from normal operating data. In practical use, these statistics can be displayed as normalized alarm indicators for plant operators. For example, $J_{z}$ can be used to indicate operating-state deviation, $J_{x}$ can be used to indicate process-side abnormality, and $J_{y}$ can be used to indicate quality-side or performance-side abnormality. It should be noted that these indicators are designed for fault detection and process–quality interpretation, rather than complete root-cause diagnosis. Detailed root-cause localization may require additional contribution analysis, variable-level diagnosis, or process knowledge.

It should be noted that this interpretation strategy does not rely on variable contribution analysis. Instead, it focuses on the abnormal patterns of different monitoring spaces. This is consistent with the objective of process–quality monitoring, where the key issue is to determine whether an abnormal event only disturbs the process-variable space or further affects the quality-related or performance-variable space. Compared with conventional $SPE_{x}$ and $SPE_{y}$ based interpretation, the proposed $J_{x}$ and $J_{y}$ statistics are calculated from conditional residual likelihoods. Thus, the abnormality of a residual is evaluated under the current latent operating condition. This enables CR-DVCCA to distinguish whether a residual pattern is truly abnormal or merely caused by state-dependent normal fluctuation. Although the proposed interpretation strategy does not rely on variable contribution analysis, such analysis can be incorporated as an additional diagnostic layer in practical applications. After an alarm is triggered, operators may inspect the corresponding residual trends, process measurements, operating logs, and process knowledge to confirm whether the alarm is physically meaningful. A systematic variable-level contribution analysis for conditional residual likelihood statistics will be investigated in future work.

## 5. Case Studies

### 5.1. Three-Phase Flow Facility

**Figure 2:**
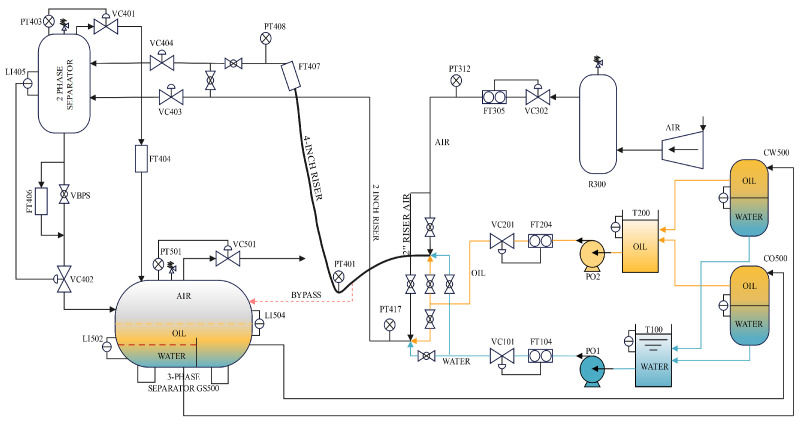
The diagram of TPFF process.

The three-phase flow facility (TPFF) is a pressurized benchmark process developed for process monitoring studies [26,29]. It is designed to feed controlled and measured flow rates of air, water, and oil into an interconnected separation system, and it has been widely used to evaluate the effectiveness of multivariate monitoring methods under realistic operating conditions and fault scenarios. As illustrated in Figure 2, the process mainly consists of a two-phase separator, a three-phase separator, and the associated pipelines and valves. In the TPFF dataset, multiple operating conditions and fault cases are provided, making it possible to assess both fault detection performance and the ability of a monitoring method to distinguish process-side and quality-side abnormalities. In this study, two representative fault cases are selected from the TPFF benchmark to evaluate the monitoring behavior of PLS, CCA, DCCA, DVCCA, and CR-DVCCA.

For the TPFF case studies, $\lambda_{y}$ and β are selected using the normal training data of the TPFF process. Since Case 1 and Case 2 are obtained from the same benchmark process and share the same process-quality variable structure, the same parameter setting was adopted for both TPFF fault cases. In practice, candidate pairs of $\lambda_{y}$ and β are evaluated, and the pair that produced the lowest converged average value of $\lambda_{y}$ on the TPFF training data is selected. As shown in Table 2, the lowest objective value was obtained when $\lambda_{y}=1.2$ and β=0.02. Therefore, these values were used for both TPFF cases. The auxiliary reconstruction weight is set to $\gamma_{\mathrm{recon}}=0.25$. Further, the encoder consists of two hidden layers with widths 64 and 32, followed by separate mean and log-variance heads, and the latent dimension is fixed to l=4. The process-side decoder uses a 4-32-64 multilayer perceptron, the quality-side decoder uses a 4-16 multilayer perceptron, and the process-side residual covariance is modeled using a low-rank Gaussian structure with residual rank $k_{x}=1$. All hidden layers employ the SiLU activation function. The model is trained using the Adam optimizer with learning rate $1\times 10^{-3}$, weight decay $1\times 10^{-5}$, batch size 128, and 80 training epochs. The random seeds are fixed to {42,52,62}, and the median-performing run among the three seeds is reported to reduce the influence of favorable initialization. For the nonlinear baselines, DCCA and DVCCA use the same optimizer setting and the same encoder–decoder backbone whenever applicable, so that the performance differences mainly reflect the residual modeling strategy rather than substantially different training budgets or manually favored hyperparameters.

#### 5.1.1. Case 1: Air Line Blockage

For Case 1, a total of 4467 test samples are collected. The air line blockage fault is introduced from sample 657 and persists until sample 3775, after which the fault is removed and the process gradually returns to the normal operating condition. To better characterize the fault evolution, the fault interval is further divided into a low-risk stage (samples 657–2995) and a high-risk stage (samples 2996–3775). This case is therefore suitable for examining whether a monitoring method can track the gradual propagation of an initially weak process disturbance into a more significant abnormal condition.

**Figure 3:**
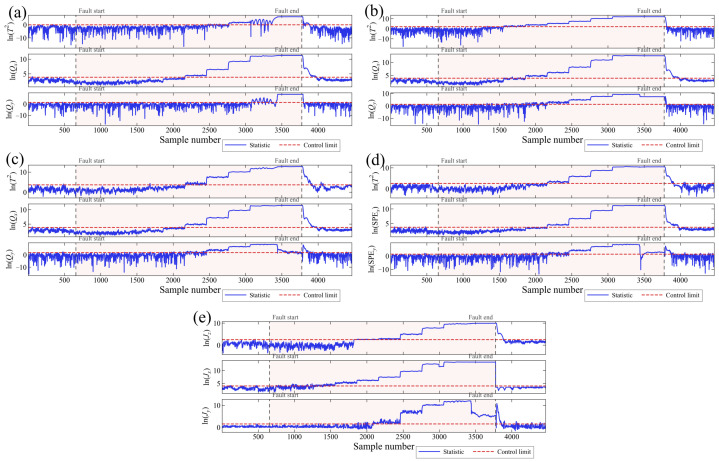
Fault detection results of air line blockage in the TPFF process using (**a**) PLS, (**b**) CCA, (**c**) DCCA, (**d**) DVCCA, and (**e**) CR-DVCCA. The blue solid curves represent the monitoring statistics, and the red dashed lines denote the corresponding control limits. The monitoring statistics are plotted on a logarithmic scale for better visualization. The shaded region between the two vertical dashed lines indicates the fault interval, with the labels “Fault start” and “Fault end” marking the beginning and removal of the fault, respectively.

The results of different algorithms are shown in Figure 3. It indicates that CR-DVCCA provides a clearer description of the fault propagation pattern than the competing methods. Specifically, as shown in Table 3, the FDR values of $J_{z}$, $J_{x}$, and $J_{y}$ are 62.69%, 82.02%, and 55.74%, respectively, while the corresponding FAR values are 0.76%, 1.52%, and 0.00%. Among the three statistics, $J_{x}$ is the most sensitive to the blockage fault and achieves a substantially higher detection rate than the process-related statistics of PLS, DCCA, and DVCCA, while still keeping the false alarm rate below 2%. In contrast, $J_{y}$ remains more conservative during normal operation, which is reflected by its zero false alarm rate. These results suggest that, in this fault scenario, the abnormality first appears in the process-related subspace and is then propagated to the quality-related part of the system. Such a response pattern is consistent with the physical nature of an air line blockage, where the disturbance initially affects process dynamics before causing more pronounced downstream deviations. Therefore, although CR-DVCCA does not give the highest detection rate for every single statistic, it offers a more interpretable and better-balanced monitoring result for this evolving fault.

#### 5.1.2. Case 2: Open Direct Bypass

For Case 2, a total of 4451 test samples are considered. The fault is caused by opening the direct bypass valve, which produces a sustained deviation from the normal operating condition and is followed by a recovery period after the fault is removed. Compared with Case 1, this scenario exhibits a more persistent abnormal pattern and is therefore suitable for evaluating whether different monitoring methods can maintain high fault sensitivity while avoiding false alarms throughout a long fault duration.

**Figure 4:**
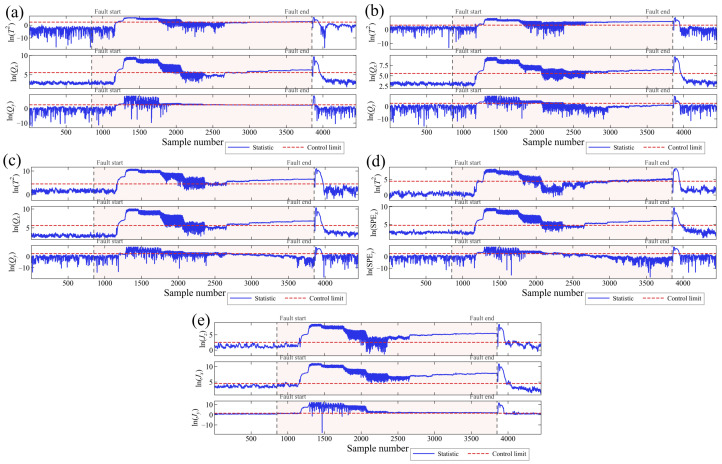
Fault detection results of Case 2 (open direct bypass) in the TPFF process using (**a**) PLS, (**b**) CCA, (**c**) DCCA, (**d**) DVCCA, and (**e**) CR-DVCCA. The blue solid curves represent the monitoring statistics, while the red dashed lines indicate the corresponding control limits. All monitoring statistics are displayed on a logarithmic scale for better visualization. The shaded region between the two vertical dashed lines denotes the fault interval, and the labels “Fault start” and “Fault end” indicate the beginning and removal of the fault, respectively.

The results of different algorithms are shown in Figure 4. It can be seen that CR-DVCCA shows the strongest overall performance in this case. Further, according to Table 3, the FDR values of $J_{z}$, $J_{x}$, and $J_{y}$ reach 86.10%, 90.87%, and 91.07%, respectively, while all three FAR values remain 0.00%. By comparison, the competing methods can achieve relatively good results for some individual statistics, but their overall detection performance is still less balanced, especially on the quality-related index. The results suggest that CR-DVCCA can separate the normal and faulty operating conditions more consistently and can simultaneously maintain high sensitivity in the latent, process, and quality subspaces. In other words, for the open direct bypass case, CR-DVCCA provides a more robust and more comprehensive monitoring performance than PLS, CCA, DCCA, and DVCCA.

### 5.2. Continuous Stirred Tank Reactor

**Figure 5:**
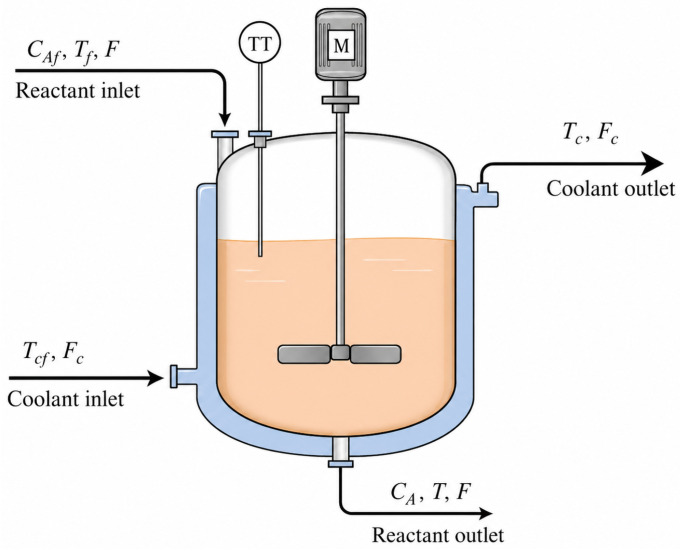
Schematic diagram of the continuous stirred tank reactor (CSTR) process, redrawn by the authors based on Ref. [30].

The continuous stirred tank reactor (CSTR) considered in this study is a simulated nonlinear exothermic reaction process [30]. As shown in Figure 5, its dynamic behavior is described by the standard material and energy balance equations

(57) $$\frac{dC_{A}}{dt}=\frac{q}{V}\left(C_{Af}-C_{A}\right)-k_{0}\exp\;\left(-\frac{E}{RT}\right)C_{A},$$

(58) $$\frac{dT}{dt}=\frac{q}{V}\left(T_{f}-T\right)+\frac{-\Delta H}{\rho C_{p}}k_{0}\exp\;\left(-\frac{E}{RT}\right)C_{A}+\frac{UA}{V\rho C_{p}}\left(T_{c}-T\right),$$

where $C_{A}$ and *T* denote the reactor concentration and reactor temperature, respectively, $C_{Af}$ and $T_{f}$ are the feed concentration and feed temperature, $T_{c}$ is the coolant temperature, *q* is the volumetric flow rate, *V* is the reactor volume, $k_{0}$ is the pre-exponential factor, E/R is the normalized activation energy term, ΔH is the reaction enthalpy, ρ is the liquid density, $C_{p}$ is the heat capacity, and UA is the overall heat transfer coefficient. Based on these process states, eight process-related variables and one quality-related variable are constructed for monitoring, where the outlet concentration is treated as the quality index.

A total of 1200 test samples are considered. The fault is introduced from sample 200 to sample 899 by imposing a gradual bias on the measured coolant temperature, and the process then enters the recovery stage from sample 900 to sample 1199. Since the disturbance is introduced as a measurement bias in the coolant-temperature variable, this case is suitable for examining whether the monitoring method can distinguish a process-side measurement abnormality from a confirmed quality-side residual alarm.

For the CSTR case, $\lambda_{y}$ and β are selected separately because the CSTR process has different variable dimensions, nonlinear dynamics, and process-quality relationships from the TPFF benchmark. The same selection principle is used: candidate pairs of $\lambda_{y}$ and β are tested on the normal CSTR training data, and the pair minimizing the converged average value of $J_{\mathrm{CR}-\mathrm{DVCCA}}$ is adopted. As shown in Table 4, the lowest objective value was obtained when $\lambda_{y}=0.8$, β=0.03. Therefore, these values are adopted in the CSTR experiment. The auxiliary reconstruction weight is set to $\gamma_{\mathrm{recon}}=0.20$. The process-side input dimension is 8 and the quality-side input dimension is 1. The CR-DVCCA encoder used a lighter two-hidden-layer architecture with widths 32 and 16, the latent dimension is set to l=4, the process-side decoder uses a 4-16-32 structure, and the quality-side decoder uses a 4-8 structure. The conditional process-side residual covariance is modeled with residual rank $k_{x}=1$. All hidden layers again use the SiLU activation function. The model is trained using Adam with learning rate $1\times 10^{-3}$, weight decay $1\times 10^{-5}$, batch size 64, and 100 epochs.

**Figure 6:**
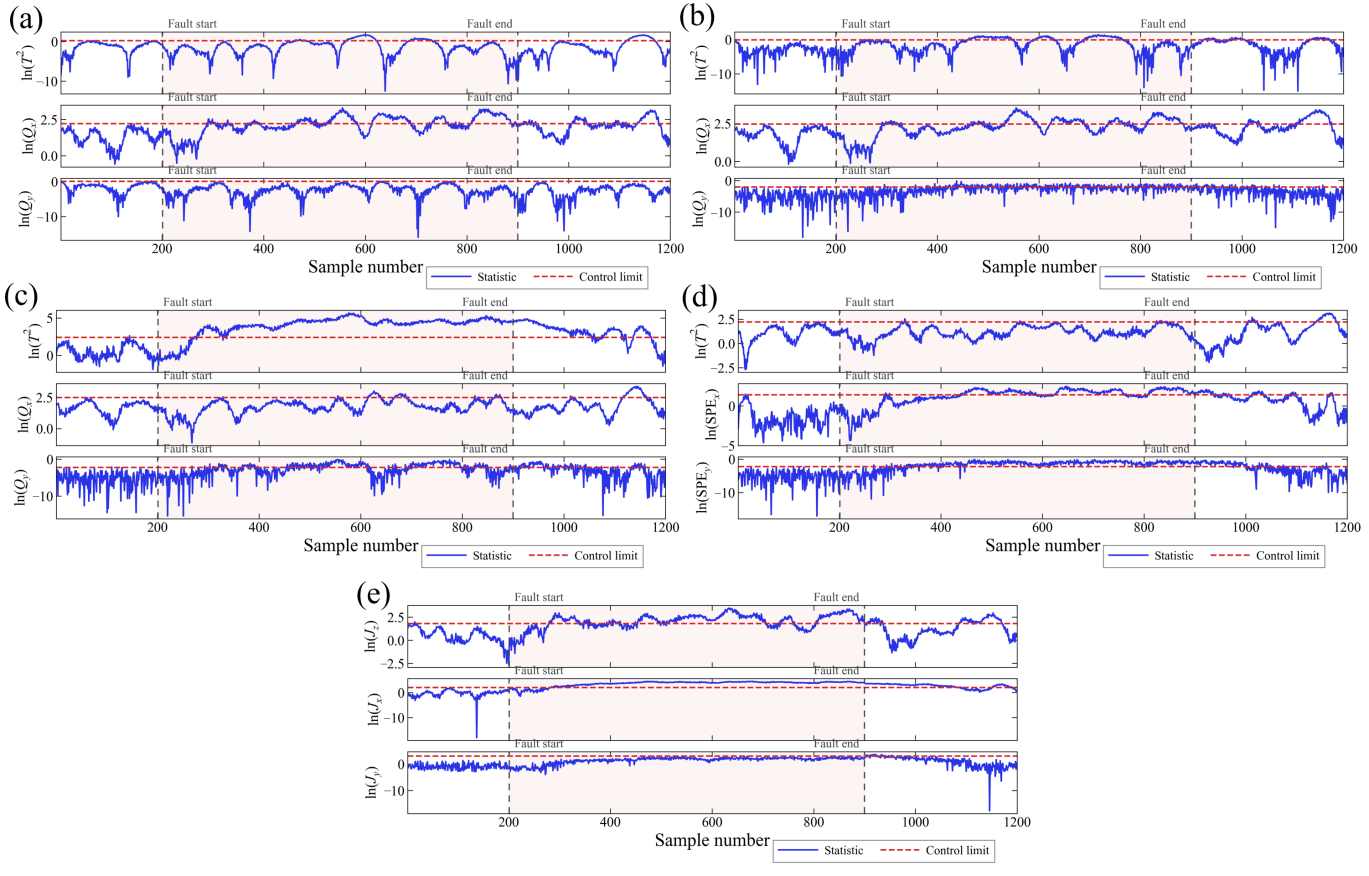
Fault detection results of the CSTR case using (**a**) PLS, (**b**) CCA, (**c**) DCCA, (**d**) DVCCA, and (**e**) CR-DVCCA. The blue solid curves represent the monitoring statistics, while the red dashed lines indicate the corresponding control limits. All monitoring statistics are displayed on a logarithmic scale for better visualization. The shaded region between the two vertical dashed lines denotes the fault interval, and the labels “Fault start” and “Fault end” indicate the beginning and removal of the fault, respectively.

The monitoring results are shown in Figure 6 and Table 5. It can be seen that CR-DVCCA provides the most interpretable monitoring behavior for this fault scenario. The FDR values of $J_{z}$, $J_{x}$, and $J_{y}$ are 66.29%, 88.29%, and 0.00%, respectively, while the corresponding FAR values are 1.50%, 0.00%, and 0.00%. In particular, the high detection rate of $J_{x}$ indicates that the process-related disturbance is effectively captured, whereas the suppressed response of $J_{y}$ confirms that the fault does not significantly propagate to the quality-related subspace. By comparison, the employed competing methods still produce either weaker process-side sensitivity or non-negligible quality-side responses. It is also worth noting that DCCA shows a large difference between its latent statistic and process-side residual statistic in this case. Specifically, the FDR of the DCCA $T^{2}$ statistic is 89.14%, whereas the FDR of $Q_{x}$ is only 9.14%. This difference is mainly due to the different meanings of the two statistics. The $T^{2}$ statistic measures the deviation of the nonlinear latent correlated features, while $Q_{x}$ measures the residual variation not captured by the process-side representation. For the coolant-temperature sensor bias, the fault introduces a systematic shift in the process measurement channel, which can strongly affect the latent correlated representation learned by DCCA. However, this shift may still be largely located along the learned nonlinear process manifold or partially absorbed by the DCCA feature mapping, and therefore does not necessarily produce a large process-side residual energy. Thus, the high $T^{2}$ FDR and low $Q_{x}$ FDR of DCCA are not contradictory, but indicate that the two statistics are sensitive to different aspects of the fault. In contrast, the proposed CR-DVCCA evaluates the process-side residual using the conditional residual likelihood $J_{x}$, which provides a more direct and sensitive measure of the coolant-temperature measurement abnormality in this case. Therefore, it suggests that CR-DVCCA is better able to preserve process-quality discrimination and to provide clearer fault detection for the CSTR process.

In this case, the imposed fault is a gradual bias added to the measured coolant temperature, rather than a direct physical change in the actual coolant temperature supplied to the reactor. Therefore, the disturbance mainly appears as a process-side measurement abnormality. Although the outlet concentration is physically coupled with the reactor thermal behavior through the nonlinear reaction kinetics, the simulated fault does not directly alter the underlying concentration dynamics. Consequently, the quality-side residual statistic $J_{y}$ remains below its control limit during the fault interval. The FDR of $J_{y}$ being 0.00% should therefore be interpreted as the absence of a confirmed quality-side residual alarm under the adopted threshold, rather than as evidence that the quality variable has no numerical fluctuation.

## 6. Conclusions

This study proposed CR-DVCCA for nonlinear industrial process monitoring with explicit conditional residual modeling. By replacing fixed squared residual statistics with state-dependent residual likelihoods, the method provides a probabilistic measure of abnormality that better reflects operating-condition variations and residual coupling. The latent statistic and the two conditional residual statistics jointly support fault detection and process–quality interpretation. Case studies on the three-phase flow facility and the CSTR process demonstrate that CR-DVCCA achieves more balanced monitoring performance than several representative methods, particularly in identifying process-side disturbances and quality-related fault propagation. The results confirm that conditional residual modeling can enhance both detection accuracy and interpretability in complex industrial systems. Future work may further extend the framework to dynamic temporal modeling and variable-level fault contribution analysis. In future work, CR-DVCCA will be further evaluated on larger-scale benchmark processes, such as the Tennessee Eastman Process, to examine its scalability and generalization ability under more diverse fault scenarios.

## Figures and Tables

**Table 1:** Fault interpretation rules based on CR-DVCCA monitoring statistics.

Monitoring Pattern	Interpretation
$J_{z}\leq \psi_{J_{z}}$, $J_{x}\leq \psi_{J_{x}}$, $J_{y}\leq \psi_{J_{y}}$	Normal operating condition
$J_{z}>\psi_{J_{z}}$	Deviation from the normal latent operating space
$J_{x}>\psi_{J_{x}}$, $J_{y}\leq \psi_{J_{y}}$	Process-related but quality-unrelated abnormality
$J_{y}>\psi_{J_{y}}$	Quality-related or performance-related fault
$J_{x}>\psi_{J_{x}}$, $J_{y}>\psi_{J_{y}}$	Fault affecting both process and quality-related spaces

**Table 2:** Grid-search results for $\lambda_{y}$ and β of TPFF cases based on the converged average training objective $J_{\mathrm{CR}-\mathrm{DVCCA}}$. Lower values indicate better fitting of the normal training data.

Dataset	β	$\lambda_{y}=0.8$	$\lambda_{y}=1.2$	$\lambda_{y}=1.6$
TPFF	0.01	1.084	0.963	1.018
TPFF	0.02	0.941	0.872	0.926
TPFF	0.05	1.006	0.944	0.991

**Table 3:** Comparison of FAR and FDR (%) for the two TPFF case studies.

Fault	Index	PLS			CCA			DCCA			DVCCA			CR-DVCCA		
		$T^{2}$	$Q_{x}$	$Q_{y}$	$T^{2}$	$Q_{x}$	$Q_{y}$	$T^{2}$	$Q_{x}$	$Q_{y}$	$T^{2}$	${\mathrm{SPE}}_{x}$	${\mathrm{SPE}}_{y}$	$J_{z}$	$J_{x}$	$J_{y}$
Air line blockage	FDR	33.65	51.76	20.19	70.38	63.24	60.22	51.44	52.34	46.83	51.99	52.02	51.09	62.69	82.02	55.74
	FAR	0.91	0.76	0.46	0.91	0.76	0.61	0.76	0.76	0.91	0.46	0.91	0.91	0.76	1.52	0.00
Open direct bypass	FDR	46.18	63.11	38.62	80.94	77.64	29.66	79.71	71.84	32.46	50.58	75.84	34.62	86.10	90.87	91.07
	FAR	0.00	0.00	0.00	0.00	0.00	0.00	0.00	0.00	0.00	0.00	0.00	0.00	0.00	0.00	0.00

**Table 4:** Grid-search results for $\lambda_{y}$ and β of CSTR case based on the converged average training objective $J_{\mathrm{CR}-\mathrm{DVCCA}}$. Lower values indicate better fitting of the normal training data.

Dataset	β	$\lambda_{y}=0.6$	$\lambda_{y}=0.8$	$\lambda_{y}=1.0$
CSTR	0.01	0.758	0.693	0.731
CSTR	0.03	0.681	0.624	0.667
CSTR	0.05	0.724	0.676	0.715

**Table 5:** Comparison of FAR and FDR (%) for CSTR Case.

Fault	Index	PLS			CCA			DCCA			DVCCA			CR-DVCCA		
		$T^{2}$	$Q_{x}$	$Q_{y}$	$T^{2}$	$Q_{x}$	$Q_{y}$	$T^{2}$	$Q_{x}$	$Q_{y}$	$T^{2}$	${\mathrm{SPE}}_{x}$	${\mathrm{SPE}}_{y}$	$J_{z}$	$J_{x}$	$J_{y}$
Coolant temperature sensor bias	FDR	17.00	47.86	0.29	30.29	40.14	34.43	89.14	9.14	46.00	2.00	54.29	79.29	66.29	88.29	0.00
	FAR	0.50	0.00	0.50	0.50	0.00	0.50	0.50	0.00	0.50	0.50	0.50	0.50	1.50	0.00	0.00

## Data Availability

Data will be available on request.
